# *Journal of Clinical Medicine* Editorial 

**DOI:** 10.3390/jcm1010022

**Published:** 2012-12-18

**Authors:** Jane M. Grant-Kels

**Affiliations:** *Founding Editor-in-Chief*, Department of Dermatology, University of Connecticut Health Center, Farmington, CT 06030, USA; E-Mail: grant@uchc.edu

“Any fool can know. The point is to understand.”

—Albert Einstein

“Why yet another journal?” Because this new journal will be different in that we are not just about new information but about understanding new information. We are online, peer reviewed, with a quick turnaround time from submission to publication and without any limit regarding length! Therefore, I am honored to introduce the *Journal of Clinical Medicine* (*JCM*), which has been created to serve as a hub for disseminating new findings and discoveries in clinical medicine to clinicians and medical researchers worldwide. 

*JCM* is an international, peer-reviewed, open-access journal published online quarterly that will provide a platform for advances in health care/clinical practices, the study of direct observation of patients and general medical research. This multi-disciplinary journal is aimed at a wide audience of medical researchers and healthcare professionals. Each edition of the journal will present original research and ideas as well as reviews, communications, short notes, and book reviews involving all fields of clinical medicine. Because of our on-line format, there is no limit to publication length. We encourage our authors to publish their findings and results in as much detail as needed so that our readership will truly understand how they performed their research or worked up their patient and why. Additionally we will cover a wide spectrum of subject areas including (1) clinical trials in which we will include the design of the trial; details regarding data collection; discussion of the legal, ethical and regulator issues; and quality assurance issues; (2) medical science research; (3) epidemiolgic studies and reviews; (4) bioethic reviews and discussions; (4) in-depth reviews on preventive medicine, translational medicine, occupational health and palliative medicine; and (5) information regarding medical care and research in developing countries. Each article will undergo a rigorous peer-review process that will incorporate strict ethical policies and standards to ensure that the journal is scholarly. 

To ensure this process we have gathered a prestigious and international group for our editorial board including: Dr. Emmanuel Andrès (Department of Internal Medicine, Diabetes and Metabolic Diseases, University Hospital of Strasbourg, France), Dr. Michael Berk (School of Medicine, Deakin University, Strategic Research Centre for Psychiatry and Epidemiology, Australia), Dr. David L. Brown, (Cardiovascular diseases, Stony Brook University Medical Center, New York, NY, USA), Dr. Edzard Ernst (Complementary Medicine, Peninsula Medical School, Devon, UK), Dr. Bronwen A. Evans (Department of Child Health, School of Medicine, Cardiff University, Cardiff, UK), Dr. Nuri B. Farber (Department of Psychiatry, Washington University, St. Louis, MO, USA), Dr. Kathleen M. Gillespie (Diabetes and Metabolism, Southmead Hospital, Bristol, UK), Dr. Michael R. Hamblin (Department of Dermatology, Harvard Medical School, Wellman Center for Photomedicine, Massachusetts General Hospital, Boston, MA, USA), Dr. David T. Harris (Department of Immunobiology, Arizona Health Sciences Centre, University of Arizona, Tucson, AZ, USA), Dr. Anthony J. Lembo (Gatrointestinal diseases, Department of Medicine, Beth Israel Deaconess Medical Center, Boston, MA, USA), Dr. Laxmaiah Manchikanti (Pain Management Center, Anesthesiology and Perioperative Medicine, University of Louisville, Paducah, KY, USA), Dr. Pier Mannuccio Mannucci (Hematology, IRCCS Cà Granda Maggiore Policlinico Hospital Foundation, Milano, Italy), Dr. Hans-Jürgen Möller (Department of Psychiatry, University of Munich, Munich, Germany), Dr. Guido Nikkhah (Department of Stereotactic and Functional Neurosurgery, University Medical Center Freiburg Neurocenter, Freiburg, Germany), Dr. Julia Polak (Imperial College Tissue Engineering Centre, Imperial College London, London, UK), Dr. Parminder Raina (Geriatrics, McMaster University, Hamilton, Ontario, Canada), Dr. Russel J. Reiter (Department of Cellular & Structural Biology, The University of Texas Health Science Center, San Antonio, TX, USA), Dr. Mark S. Sands (Washington University School of Medicine, Departments of Medicine and Genetics, St. Louis, MO, USA), Dr. Evan Y. Snyder (Sanford-Burnham Medical Research Institute, Department of Pediatrics, University of California San Diego, La Jolla, CA, USA), Dr. David F. Wozniak (Department of Psychiatry, Washington University School of Medicine, St. Louis, MO, USA) and Dr. Xiao Yan Zhong (Women’s Health, Department of Biomedicine, University Hospital Basel, Basel, Switzerland).

Welcome! We invite you to submit your articles and learn along with us as we undertake this new journal. 

